# Mitochondrial Impairment in Unloaded Postural Muscle: Mechanisms Driving Loss of Muscle Function and Mass

**DOI:** 10.3390/antiox15030277

**Published:** 2026-02-24

**Authors:** Kristina A. Sharlo, Timur M. Mirzoev, Boris S. Shenkman

**Affiliations:** Myology Laboratory, Institute of Biomedical Problems RAS, Moscow 123007, Russia; tmirzoev@yandex.ru (T.M.M.);

**Keywords:** skeletal muscle, mitochondria, mechanical unloading, hindlimb suspension, dry immersion, muscle atrophy, ROS, calcium handling, ATP, mitokines

## Abstract

Mechanical unloading of skeletal muscle triggers various signaling alterations that result in muscle atrophy and weakness. Mitochondria are essential to muscle health, acting not only as energy suppliers but also as central mediators of molecular regulation. Mitochondrial activity, content, and dynamics are tightly controlled by multiple signaling pathways; conversely, mitochondria-derived messengers, such as reactive oxygen species (ROS), ATP, and mitokines, are involved in the regulation of nearly all aspects of muscle signaling. During mechanical unloading, altered muscle activity leads to mitochondrial dysfunction. However, the initial triggers, underlying mechanisms, and full consequences of this dysfunction remain poorly understood. Nevertheless, mitochondria-targeted therapies have emerged as a promising strategy for mitigating unloading-induced muscle impairments. In this review, we summarize current data regarding the characteristics, causes, and outcomes of unloading-induced mitochondrial dysfunction, specifically focusing on muscle atrophy and functional decline. We highlight novel findings regarding the roles of mitokines and mitochondrial calcium overload, propose a new hypothesis to explain the biphasic dynamics of ATP accumulation during slow-type muscle unloading, and describe emerging therapeutic strategies to counteract these mitochondrial impairments.

## 1. Introduction

The significance of skeletal muscles cannot be overstated. Constituting a large proportion of total body mass [1], skeletal muscles enable locomotion and the maintenance of posture, support respiration and thermoregulation, and play central roles in carbohydrate metabolism and endocrine function [2,3]. The loss of skeletal muscles may be related to metabolic impairments, including insulin resistance and type II diabetes, which can increase the risks of morbidity and mortality [4]. Therefore, the maintenance of skeletal muscle mass and function is imperative for overall health and quality of life [5,6]. However, under conditions of prolonged mechanical unloading/disuse (such as bedrest, limb immobilization, mechanical ventilation, and weightlessness) skeletal muscles (especially those that contain a large proportion of oxidative fibers and carry out postural/antigravity function) undergo significant structural and functional alterations resulting in reductions in muscle mass (atrophy), strength, and fatigue resistance [7,8]. Moreover, mechanical unloading elicits substantial alterations in the metabolic profile of postural muscle fibers: oxidative fibers (expressing myosin heavy chain (MyHC) I and IIa isoforms, resistant to fatigue, rich in mitochondria and rely on aerobic respiration) transform to glycolytic fibers (expressing MyHC IIx and IIb isoforms, more fatigable, contain fewer mitochondria and primarily rely on anaerobic glycolysis) [9]. In addition to fiber atrophy and the loss of force, it has been demonstrated that mechanical unloading due to weightlessness is able to reduce the ability of the rat soleus (slow-type, oxidative muscle) to oxidize fats and increases the utilization of muscle glycogen [9]. This substrate change leads to an increased rate of fatigue [9]. Interestingly, a similar transition from slow-type (oxidative) to fast-type (glycolytic) fibers along with decreased mitochondrial quantity and quality was observed in humans with mitochondrial myopathy [10]. Thus, mitochondrial impairments have the capacity to modify the structure and function of skeletal muscles, resulting in alterations in the predominant energy sources utilized, augmented loss of oxidative capacity, and subsequent muscle weakness. Central to these structural and metabolic shifts is the emergence of mitochondrial calcium dysregulation as a unifying mechanism. While mechanical unloading triggers a cascade of deleterious events, the disruption of mitochondrial calcium handling acts as a primary orchestrator that links mechanical disuse to cellular dysfunction. Thus, calcium homeostasis in skeletal muscle provides a coherent framework for understanding how mitochondrial failure initiates and sustains the atrophic process under unloading conditions.

More than 60 years ago, Carafoli et al. [11,12] (1962, 1964) first reported that mitochondrial dysfunction might contribute to skeletal muscle atrophy. They showed that three days of disuse following denervation of the pigeon breast muscle caused a marked reduction in mitochondrial content, together with decreased cytochrome oxidase activity and impaired oxidation of α-ketoglutarate, L-glutamate, and pyruvate. Importantly, these mitochondrial alterations appeared before any morphological signs of muscle atrophy. The authors suggested that defective energy-transduction mechanisms may play a key role in initiating the atrophic process and speculated that reduced ATP supply from aerobic metabolism could limit protein biosynthesis in denervated muscle [11,12]. To date, mitochondrial dysfunction is considered a key factor contributing to disuse-induced skeletal muscle atrophy. Mechanisms associated with mitochondria-related induction of inactivity-induced muscle atrophy include the generation of ROS, disturbances in mitochondrial dynamics (fission and fusion), decreased mitochondrial biogenesis, impaired regulation of autophagy, and apoptosis [13]. Indeed, skeletal muscle health is closely tied to the optimal functioning of mitochondria, which form an interconnected network with the sarcoplasmic reticulum and sarcolemma [14]. Apart from their indispensable role in the aerobic synthesis of ATP, mitochondria are vital for a variety of other important processes, such as ROS production, calcium handling and apoptosis [15,16].

The most effective method for modeling mechanical unloading of skeletal muscles in laboratory rodents (mice and rats) is hindlimb suspension/unloading (HS/HU) [17,18], which leads to significant skeletal muscle atrophy, particularly in slow-type muscles, such as the soleus muscle [19]. The HS model involves the rodent being suspended by the tail, which prevents the hind legs from bearing any weight while the animals support their weight on the front legs. In humans, mechanical unloading of skeletal muscles can be achieved by using ground-based analogs for spaceflight such as head-down tilt and supine bedrest, dry immersion, and unilateral lower limb suspension/immobilization [20].

While several recent reviews on mitochondrial dysfunction in different forms of muscle atrophy have focused mainly on altered mitochondrial turnover and the effects of ROS on protein-turnover pathways [13,21,22,23], the present review concentrates on how mitochondria regulate myoplasmic Ca^2+^ during mechanical unloading and how disrupted Ca^2+^ handling, in turn, compromises mitochondrial health. We also highlight recent findings on the crosstalk between mitochondria, cellular ATP dynamics, and mitokines. Finally, we discuss promising therapeutic strategies to reverse unloading-induced mitochondrial impairment and suggest avenues for future research.

## 2. The Impact of Mechanical Unloading on Mitochondrial State in Skeletal Muscle

### 2.1. Mitochondrial Network Structure

In 1978, it was demonstrated that mitochondria in skeletal muscle constitute an extensive branched network capable of energy transport throughout the system [24]. In skeletal muscle, mitochondria are categorized into two distinct subpopulations: subsarcolemmal (situated near the plasma membrane) and intermyofibrillar (located in direct proximity to the myofibrils). More recent studies describe subsarcolemmal mitochondria as being located adjacent to blood capillaries or myonuclei, suggesting more precise terminology for these populations, such as “paravascular” or “paranuclear” mitochondria [25,26]. In 2015, scanning electron microscopy revealed additional subgroups of intermyofibrillar mitochondria: I-band mitochondria, fiber-parallel mitochondria, and cross-fiber connection mitochondria [27]. Nonetheless, subsarcolemmal and intermyofibrillar mitochondrial populations differ in protein composition and, presumably, in their functional roles. It has been demonstrated that intermyofibrillar mitochondria exhibit a lower rate of proton leak [28,29], while subsarcolemmal mitochondria generate higher levels of peroxide and other reactive oxygen species (ROS) [30]. A study utilizing a combination of respirometry and proteomic analysis revealed that the rate of ATP synthesis in intermyofibrillar mitochondria is three times higher than in the subsarcolemmal population. Furthermore, proteomic profiling of these two subpopulations from the red portion of the rat gastrocnemius muscle indicated that intermyofibrillar mitochondria possess higher concentrations of protein synthesis components, respiratory chain proteins, and the calcium transporter VDAC (voltage-dependent anion channel) located in the mitochondrial outer membrane [31]. A subsequent study utilizing electron microscopy techniques demonstrated that the ratio of ATP synthase to respiratory chain complex IV is significantly higher in intermyofibrillar mitochondria than in the subsarcolemmal population [27]. Furthermore, that study examined and visualized the skeletal muscle mitochondrial network, revealing the presence of electron-dense contact sites between the two mitochondrial subpopulations. These contacts facilitate the redistribution of the proton gradient across the mitochondrial network. Another study investigated the response of distinct mitochondrial subpopulations to depolarization-induced calcium signaling in isolated *m. longissimus dorsi* fibers. In subsarcolemmal mitochondria, this signaling triggered a decrease in the proton gradient, whereas intermyofibrillar mitochondria exhibited a temporally delayed increase in the proton gradient [32]. This research also corroborated the findings of Glancy et al. (2015) [27] regarding the elevated concentration of complex IV at the fiber periphery and provided data indicating that the mitochondrial calcium uniporter (MCU)-inhibitory protein, MICU1, is localized exclusively in subsarcolemmal mitochondria, which may reduce their calcium sensitivity compared to intermyofibrillar mitochondria.

Based on these and other experimental findings, a hypothesis was formulated regarding the distinct roles of mitochondrial subpopulations in meeting the energetic demands of muscle during contraction. It is proposed that during muscle contraction, electron transport chain (ETC) enzymes are activated in the subsarcolemmal mitochondria, where they consume oxygen and generate a proton gradient. This gradient is then transmitted to the intermyofibrillar mitochondria, which synthesize ATP in close proximity to its primary consumers—the myofibrils [33].

In the context of the above, it is pertinent to consider the morphological changes occurring across various models and durations of mechanical unloading. Unfortunately, our literature search failed to identify data regarding mitochondrial volume density measurements for durations of less than 14 days. Following a 14-day spaceflight, a slight decrease in mitochondrial volume density was observed in the subsarcolemmal region of the soleus muscle in rhesus macaques. In contrast, the vastus lateralis exhibited a significant reduction in mitochondrial volume density in both the subsarcolemmal and central zones [34]. Following 60 days of bed rest, a significant decrease in mitochondrial volume density was observed in the subsarcolemmal zone of human soleus muscle fibers [35]. Furthermore, after 120 days of head-down tilt bed rest, mitochondrial volume density declined in both the central and subsarcolemmal zones [36]. In rats, five weeks of hindlimb unloading resulted in a reduction in subsarcolemmal mitochondrial volume density coupled with an increase in intermyofibrillar mitochondrial volume density, while total volume density remained unchanged [36]. The authors of that study proposed that pronounced soleus fiber atrophy masked the proportional decrease in absolute mitochondrial volume density. The mechanisms underlying the more pronounced decline in mitochondrial volume density within the subsarcolemmal zone remain elusive. Given that subsarcolemmal mitochondria exhibit both a higher rate of ROS production and a greater sensitivity to oxidative stress, it can be hypothesized that this reduction is associated with accelerated ROS-induced damage to this subpopulation, followed by mitophagy. Considering the role of subsarcolemmal mitochondria in supplying the proton gradient to intermyofibrillar mitochondria, it is plausible that the decrease in subsarcolemmal mitochondrial volume density negatively impacts the skeletal muscle’s capacity to meet the energetic demands of contraction during functional unloading and throughout the early recovery period.

It should be specifically noted that in skeletal muscle, mitochondrial complexes I–IV are capable of forming supramolecular assemblies, or supercomplexes. This formation enhances the efficiency of the electron transport chain (ETC) and reduces ROS generation by maintaining an optimal stoichiometric ratio between the complexes and decreasing the diffusion distance of electron carriers [37]. It has been observed that after four months of endurance training, an increased number of mitochondrial supercomplexes in elderly individuals correlated with elevated oxygen consumption in permeabilized muscle fibers [37]. Furthermore, cytochrome c oxidase assembly factor 1 (COX7RP) knockout mice, which lack the protein responsible for stabilizing supercomplexes, exhibited reduced physical activity and impaired thermogenesis in response to cold stress, whereas mice overexpressing this protein showed enhanced endurance [38]. Evidence suggests that the assembly of supramolecular complexes depends on the morphology of mitochondrial cristae [39] as well as the cardiolipin content in the inner mitochondrial membrane [40]. Additionally, optic atrophy 1/mitochondrial dynamin-like 120 kDa protein (OPA1) plays a critical role in supercomplex formation [41]. Notably, a downregulation of OPA1 expression has been demonstrated in the soleus muscle under conditions of rodent hindlimb unloading [42]. To date, we have identified no studies describing the state of supercomplexes under mechanical unloading. Nevertheless, it is evident that the organizational state of ETC supercomplexes must significantly influence mitochondrial function, including the capacity to meet muscle energy demands during unloading and the regulation of mitochondrial ROS production.

### 2.2. Mitophagy

Data regarding mitophagy levels under conditions of skeletal muscle unloading remain largely contradictory. In most instances, researchers infer mitophagy activity based on the mRNA expression levels of various components within the autophagy–mitophagy pathway. Frequently, different components of this signaling pathway exhibit divergent changes; moreover, they are involved not only in mitophagy but also in other catabolic signaling processes. Nevertheless, a body of evidence suggests that mitophagy is inactivated in rat soleus muscle during hindlimb unloading. This inactivation has been demonstrated both through the analysis of mitophagy marker expression and via the colocalization of the lysosomal-associated membrane protein (LAMP1) with the translocase of the outer mitochondrial membrane 20 (TOMM20) protein on muscle cryosections using fluorescent labeling [43]. In contrast, mitophagy activation has been observed in fast-twitch muscles during both early and late stages of immobilization [44,45].

In humans, neither short-term nor prolonged head-down tilt bed rest induces significant changes in mitophagy levels within the vastus lateralis muscle [46,47]. Following 6 days of dry immersion, the expression of mitophagy regulators in the human soleus muscle was found to be downregulated [48]. During the 21-day immersion, mitophagy-associated mRNA transcripts in the human soleus muscle exhibited multidirectional changes [49].

Consequently, current data on mitophagy under mechanical unloading do not provide a definitive answer as to whether the reduction in mitochondrial density is linked to mitophagy activation. It appears that mitophagy is differentially regulated depending on the unloading model and muscle fiber type. Synthesizing these findings, we propose that mitophagy should be viewed primarily as a compensatory quality control mechanism rather than the primary cause of mitochondrial loss. While its inactivation may lead to the accumulation of dysfunctional mitochondria, its excessive activation likely represents a secondary response to severe mitochondrial damage. Thus, rather than being a primary driver of atrophy, altered mitophagy appears to be a marker of the muscle’s attempt to maintain energetic homeostasis under mechanical unloading.

### 2.3. Mitochondrial Dynamics

In all identified studies, data regarding mitochondrial dynamics (i.e., coordinated cycles of fission and fusion) under conditions of mechanical unloading were limited to the assessment of mRNA expression or protein levels of various fusion and fission regulators; no direct measurements of mitochondrial fusion or fission rates were conducted. As early as the first day of hindlimb unloading, a decrease in mitofusin-1 mRNA expression is observed in the soleus muscle, while other transcripts regulating mitochondrial dynamics remain unchanged [50]. Most studies involving rodent hindlimb unloading for durations of 7, 14, and 28 days have reported a reduction in the expression of both mitochondrial fusion and fission components in the soleus muscle [51,52]. On the third day of unloading, the mouse gastrocnemius muscle exhibited a decline in OPA1 and mitofusin-1 expression, but not mitochondrial fission 1 protein (Fis1), alongside an increase in dynamin-related protein 1 (Drp1) phosphorylation, suggesting a predominance of mitochondrial fission over fusion during the early stages of functional unloading [42].

In humans, a 10-day period of bed rest resulted in a downward trend in the expression of mitofusins-1 and -2, as well as phospho-Drp1, in the vastus lateralis muscle [53]. Six days of dry immersion led to a reduction in the mRNA levels of both fusion and fission regulators in the human soleus muscle [48]. It appears that the earliest stages of functional unloading in both fast-twitch and slow-twitch muscles are characterized by a shift toward mitochondrial fission (driven by the downregulation of fusion components), followed by a subsequent decline in the overall levels of both mitochondrial fusion and fission.

### 2.4. Mitochondrial Biogenesis

A reduction in mitochondrial biogenesis markers under mechanical unloading is observed across the majority of experiments, regardless of the duration, unloading model, or muscle type (slow-twitch or fast-twitch). In the soleus muscle, regulators of mitochondrial biogenesis exhibit a decrease in mRNA expression as early as after 24 h of rat hindlimb unloading [50]. By days 3–7 of hindlimb unloading, rat soleus muscle exhibits a reduction in peroxisome proliferator-activated receptor gamma coactivator 1-alpha (PGC-1α) protein levels, as well as a decline in respiratory chain complex IV subunit I, the mitochondrial membrane protein TOMM20, and the mitochondrial DNA to nuclear DNA copy number ratio [54]. Similarly, in the mouse vastus lateralis, a 3-day unloading period leads to a downregulation of PGC-1α and other mitochondrial biogenesis regulators [55].

A decrease in the mRNA expression of mitochondrial biogenesis regulators is also observed in the human soleus muscle on days 3 and 6 of dry immersion, and in the vastus lateralis by day 6 [48,56].

In human vastus lateralis, 5 days of head-down tilt bed rest resulted in a reduction in mRNA transcripts associated with mitochondrial biogenesis and function [57], although no changes were detected in biogenesis or dynamics components at the protein level [47]. However, after 60 days of bed rest, a significant decrease in mRNA transcripts associated with mitochondrial biogenesis was observed in the vastus lateralis muscle [58].

Given the data on mitophagy under mechanical unloading—which suggest its inactivation rather than activation—it can be hypothesized that the suppression of mitochondrial biogenesis is the primary driver of reduced mitochondrial density in slow-twitch muscles. Nevertheless, several studies suggest that, at least in slow-twitch muscles, the decline in mitochondrial biogenesis may decelerate after 14 days of unloading. For instance, after 28 days of hindlimb unloading in rats, no reduction was found in respiratory chain complex IV components or mitochondrial transcription factor A (TFAM) levels [52]. In human soleus muscle following 21 days of dry immersion, PGC-1α and cytochrome c oxidase 1 (COX-1) protein levels did not differ from pre-immersion values; however, transcriptomic analysis revealed that a substantial number of transcripts associated with mitochondrial biogenesis and mitochondrial ribosomal protein synthesis were significantly downregulated [49]. In mouse gastrocnemius muscle after 14 days of unloading, no decrease in PGC-1α protein levels was detected, despite a decline in other biogenesis components [59]. The potential mechanisms underlying these observations will be discussed further.

## 3. The Effect of Mechanical Unloading on Mitochondrial Functions and Calcium Handling in Skeletal Muscle

Studies investigating mitochondrial enzymes under conditions of muscle unloading typically employ several distinct methodological approaches to assess enzymatic activity. In earlier research, the most common method involved determining the enzymatic activity of respiratory chain components or the Krebs cycle within muscle lysates or on transverse muscle cryosections. Within this methodology, the fluorescent signal from the reaction’s end product is normalized to the fiber cross-sectional area. Another widely utilized approach for evaluating respiratory chain enzyme activity is respirometry, which measures the amount of oxygen consumed by a sample in a specific buffer solution during the sequential addition of uncouplers. Respirometry analysis is performed using either isolated mitochondria or skinned muscle fibers, with the resulting data typically normalized to tissue weight, and occasionally to lyophilized weight.

It should be noted that mitochondrial enzyme activity, the concentration of specific mitochondrial enzymes, and mitochondrial density are often treated as synonymous or highly interdependent parameters in the literature. However, mitochondrial activity within muscle tissue is strictly regulated to meet the energetic demands of muscle contraction; under different activity regimes, mitochondrial performance can vary by several orders of magnitude while maintaining a constant level of mitochondrial density.

### 3.1. Mitochondrial Oxygen Consumption

Analysis of mitochondrial oxygen consumption in the mouse soleus muscle during 3 and 7 days of hindlimb unloading revealed a significant decrease in this parameter across all investigated electron transport chain (ETC) complexes [60]. Similar findings were reported following 3 and 6 days of dry immersion in human soleus muscle [56,61]. Conversely, two distinct experiments involving 7-day rat hindlimb unloading demonstrated a significant increase in oxygen consumption by several ETC complexes in the presence of uncouplers [43,62]. Earlier studies indicated a reduction in oxygen consumption by skinned rat soleus muscle fibers after 3, 7, and 14 days of unloading; however, it should be noted that this study utilized buffer solutions with high calcium concentrations, in contrast to the aforementioned research which employed calcium-free solutions. Furthermore, no changes in tissue oxygen consumption were detected in the soleus or gastrocnemius muscles of mice after 1, 3, or 7 days of unloading in a study that also utilized calcium-containing buffers [63]. Under conditions of head-down tilt bed rest, one study reported a significant increase in ADP-stimulated mitochondrial oxygen consumption in vastus lateralis fibers after 4 days using a calcium-free buffer [64]. In contrast, another study on the same muscle after 7 days of bed rest detected a significant decrease in ADP-stimulated respiration using a calcium-containing solution [65]. To reconcile these conflicting experimental findings, it is necessary, in our view, to examine the specific regulatory mechanisms of mitochondrial activity in skeletal muscle.

Earlier research on mitochondrial ETC enzyme activity in skeletal muscle hypothesized that ATP synthesis was regulated exclusively by ADP concentration—meaning it was driven by ATP consumption (primarily via the actomyosin complex, the sarcoplasmic/endoplasmic reticulum Ca^2+^ ATPase (SERCA), and Na^+^/K^+^-ATPase activity). Within this framework, calcium was thought to activate the ETC only indirectly by stimulating tissue-level ATP consumption However, a growing body of evidence now demonstrates that calcium activates ETC enzymes at least semi-directly (beyond just stimulating ATP demand) by activating NADH dehydrogenases (pyruvate, isocitrate, and alpha-ketoglutarate dehydrogenases); it is also capable of directly stimulating ATP synthase and complex III [27,66]. Computer simulations further suggest that the activation of ETC enzymes and the increase in muscle ATP expenditure occur in parallel through calcium-dependent regulation [67]. In addition to calcium, mitochondrial enzyme activity can be modulated by post-translational modifications, specifically phosphorylation [68], and oxidation by various ROS, primarily of mitochondrial origin [69]. It is important to note, however, that standard analytical methods assess mitochondrial enzyme activity under “resting” conditions—using constant pH and ionic composition, saturated levels of ADP and oxygen, and in the presence of reducing agents that eliminate the effects of ROS on ETC activity. Furthermore, while some respirometry protocols utilize calcium concentrations comparable to the maximum mitochondrial calcium capacity, others employ entirely calcium-free solutions. It should be noted that in the absence of calcium, skinned muscle fibers may exhibit calcium release from the sarcoplasmic reticulum (SR) due to the opening of potassium channels that fail to close in a calcium-free environment, leading to subsequent sarcolemmal depolarization [70]. Given that calcium entering from the SR directly influences intramitochondrial calcium levels [32], a significant mitochondrial calcium concentration may be maintained for a prolonged period during the assay. In our laboratory, staining of mitochondria in permeabilized fibers with Rhod-2 AM demonstrated the presence of calcium in the mitochondrial matrix after 90 min of incubation in a calcium-free MIRO5 buffer (unpublished data). This may be attributed to the calcium reserves within the SR and the close interaction between the mitochondrial calcium pool and the SR via microdomain calcium signaling. Consequently, differences in respirometry rates between control and unloaded muscle fibers may be determined by variations in intramitochondrial calcium levels or differences in the state of the SR-to-mitochondria calcium flux. In this case, if intramitochondrial calcium concentrations are equalized across all samples, the respirometry data would reflect the maximum oxygen consumption capacity of ETC enzymes—proportional to their content in the sample—rather than the actual in vivo mitochondrial oxygen consumption within the muscle.

It is important to distinguish between the “maximal oxidative capacity” measured in the ex vivo respirometry protocols and the actual in vivo mitochondrial respiration during mechanical unloading. Most current assays utilize saturating concentrations of substrates, which effectively bypasses the physiological regulatory constraints, such as fluctuating calcium microdomains and ROS-dependent modifications present in a living muscle fiber. Thus, while these data reflect the total enzymatic potential (proportional to ETC content), they may not accurately represent the true metabolic performance of the postural muscle in vivo.

### 3.2. Mitochondrial Enzyme Activity

Following 10 days of rat hindlimb unloading, a 40% reduction in citrate synthase activity and a 50% decrease in cytochrome c oxidase activity were observed in the soleus muscle; however, no decline in succinate dehydrogenase or lactate dehydrogenase activity was detected in that study [71]. Notably, enzyme activity in this experiment was normalized to milligrams of total protein. Similarly, a 5-week hindlimb unloading study in rats reported a decrease in the activity of 3-hydroxyacyl-CoA dehydrogenase and citrate synthase, but not lactate dehydrogenase, when normalized to tissue weight [72]. After 7 days of unloading, a significant reduction in citrate synthase activity was found in both the soleus and gastrocnemius muscles [73,74]. Furthermore, three weeks of unloading resulted in a decline in succinate dehydrogenase activity in the rat soleus [75]. In contrast, other research identified a substantial increase in citrate synthase activity in the rat soleus after 14 and 28 days of unloading [76]. Additionally, several studies involving hindlimb unloading and spaceflights of various durations reported an activation of succinate dehydrogenase within muscle fibers [77]. In these instances, enzyme activity was normalized to the dry weight of the muscle samples. It is worth noting that the methodological challenges encountered in the analysis of enzyme activity are analogous to those inherent in respirometry. Specifically, conditions of substrate excess and optimal parameters for pH, calcium content, inorganic phosphate, and ROS levels within the assay result in measurements that reflect the maximum enzymatic activity proportional to its concentration in the sample, rather than the actual activity of the enzyme within the muscle. Furthermore, the assessment of individual enzyme activities, much like respirometry in the presence of uncouplers, cannot reveal shifts in mitochondrial function associated with specific mitochondrial subpopulations or the structural state of supercomplexes.

Consequently, to understand how mitochondrial activity truly changes in the muscle during mechanical unloading, it is necessary to cross-reference data from mitochondrial enzyme activity assays with indirect data that reflect the in vivo state of mitochondrial functions.

### 3.3. ATP and ROS Content in Skeletal Muscle

The primary products of mitochondria in a skeletal muscle are ATP and ROS. Under conditions of mechanical unloading, there is an increase in the production of both mitochondrial ROS and ROS from other sources, specifically NADPH oxidases and xanthine oxidase. Furthermore, the excessive generation of ROS by NADPH oxidase 2 (NOX2) is capable of triggering mitochondrial ROS production [78,79]. Under physiological conditions, the primary sources of mitochondrial ROS are complexes I and III of the respiratory chain [80]. However, certain Krebs cycle enzymes are also capable of ROS generation; specifically, in addition to the succinate dehydrogenase complex, alpha-ketoglutarate dehydrogenase contributes to ROS production [81]. Under mechanical unloading, mitochondrial-derived ROS have been shown to accumulate in the rat soleus muscle as early as after 6 h of hindlimb suspension [82]. Similarly, after 24 h of denervation, Karam and colleagues (2017) detected an increase in mitochondrial ROS in the flexor digitorum brevis muscle [83]. Although denervation is not fully equivalent to hindlimb unloading, it is comparable to the initial 24 h of soleus muscle mechanical unloading since soleus muscle neuromuscular activity is dramatically reduced. Elevated ROS levels in muscle tissues during functional unloading have also been observed on days 7 and 14 of hindlimb unloading in the mouse soleus [60]. Furthermore, increased mitochondrial ROS production during respirometry analysis was demonstrated following 4 days of bed rest in the human vastus lateralis [64]. The mechanisms driving the surge in mitochondrial ROS generation remain not fully understood. Karam et al. (2017) attribute the increased mitochondrial ROS production under denervation to impaired mitochondrial calcium signaling [83]. Several other authors also suggest that the rise in mitochondrial ROS generation is linked to calcium-dependent processes within the disused skeletal muscle [84].

One signaling pathway that may be involved in mitochondrial dysfunction and subsequent excessive ROS production under disuse conditions is the Janus kinase (JAK)/signal transducer and activator of transcription 3 (STAT3) pathway. In inactive skeletal muscle, STAT3 can translocate into mitochondria and bind to one of the subunits of the respiratory complex I, resulting in increased activity of ETC and ROS production by mitochondria [85]. Indeed, in a rat model of ventilation-induced diaphragm atrophy, activation of the JAK/STAT3 pathway leads to STAT3 phosphorylation at Tyr705 and Ser727, translocation of phosphorylated STAT3 into mitochondria, and consequent mitochondrial dysfunction [86]. Treatment with a selective JAK inhibitor prevented the disuse-induced mitochondrial accumulation of phosphorylated STAT3, restored mitochondrial function, reduced ROS generation, and preserved diaphragm mass and contractile function [86]. Importantly, disuse activates the JAK/STAT signaling axis not only in the inspiratory diaphragm muscle but also in limb plantar-flexor muscles such as the plantaris [87]. It is also important to note that the cytokine interleukin-6 (IL-6) can act as an upstream activator of the JAK/STAT pathway, which plays a role in the initiation of skeletal muscle atrophy [88,89]. Our laboratory has demonstrated that HS results in a significant increase in IL-6 mRNA expression and STAT3 nuclear translocation [90]. Moreover, L-type calcium channel blockade prevented the HS-induced increase in IL-6 mRNA expression in the rat soleus muscle [90], suggesting that calcium accumulation plays an important role in regulating the IL-6/JAK/STAT signaling pathway during disuse. In addition, chronically elevated IL-6 can induce the expression of Drp-1 and Fis-1 (key proteins in mitochondrial fission) in skeletal muscle both in vitro and in vivo [91], potentially leading to mitochondrial fragmentation and reduced ATP efficiency.

In contrast to the unidirectional changes observed in ROS levels, ATP content in the myoplasm of postural muscles exhibits a non-linear dynamic during unloading. On days 1–3 of soleus muscle unloading, an accumulation of ATP within that muscle occurs [50,92], which is accompanied by a decrease in the phosphorylation level of the key skeletal muscle energy sensor, AMP-activated protein kinase (AMPK). AMPK inactivation was detected as early as several hours after the onset of soleus muscle unloading [93]. However, by day 7 of rat hindlimb unloading, ATP content and AMPK phosphorylation in the soleus do not differ from control values [94]. Conversely, by day 14 of unloading, a significant decrease in tissue ATP content is observed compared to control, accompanied by an increase in AMPK phosphorylation [94,95,96]. In certain studies, 14 days of soleus unloading did not result in an increase in AMPK phosphorylation, yet it led to the accumulation of its downstream target, phosphorylated ACC [97,98]. Moreover, the phosphorylation of AMPK in the soleus muscle remained elevated at 4, 8 and 13 weeks of unloading [99]. Under conditions of human dry immersion, a decrease in phospho-AMPK levels is observed on day 3 [100], while on day 21, there is a trend toward increased phospho-AMPK and a significant rise in the levels of the AMPK target, phospho-acetyl-CoA carboxylase (ACC) [49]. Thus, ATP levels in the unloaded postural muscle demonstrate non-linear dynamics; since ATP generation in the muscle is directly linked to mitochondrial function, it is possible that these ATP fluctuations reflect non-linear processes of mitochondrial dysfunction occurring during the course of unloading. However, not all investigations align with this pattern. For instance, while one study reported increased ATP accumulation after 14 days of rat hindlimb unloading, another found that AMPK phosphorylation was significantly downregulated in the soleus at the same time point [101,102].

Undoubtedly, ATP content in skeletal muscle is influenced both by mitochondrial synthesis and by ATP consumption. Specifically, during the early stages of functional unloading, the functions of Na^+^,K^+^-ATPase and SERCA are impaired; moreover, there is a significant reduction in the electromyographic activity of muscle fibers (a marker of myofibrillar contractile activity), which subsequently undergoes gradual recovery up to control values by day 14 of unloading [103,104,105]. Nevertheless, indirect evidence of the mitochondrial contribution to ATP accumulation during early functional unloading can be found in experiments involving the administration of metformin (an ETC complex I inhibitor) and beta-guanidinopropionic acid (a creatine kinase inhibitor). Both substances reduce the efficiency of mitochondrial ATP synthesis; their application on days 1 and 3 of rat hindlimb unloading significantly prevented ATP accumulation in the soleus muscle tissue. It is probable that mitochondrial ATP synthesis is elevated during the early stages of mechanical muscle unloading and diminished during the later stages. Importantly, both the increase and subsequent decline in ATP synthesis may be driven by calcium-dependent processes, which will be discussed in the following section. 

### 3.4. Mitochondrial Calcium Handling During Mechanical Unloading

**Figure 1:**
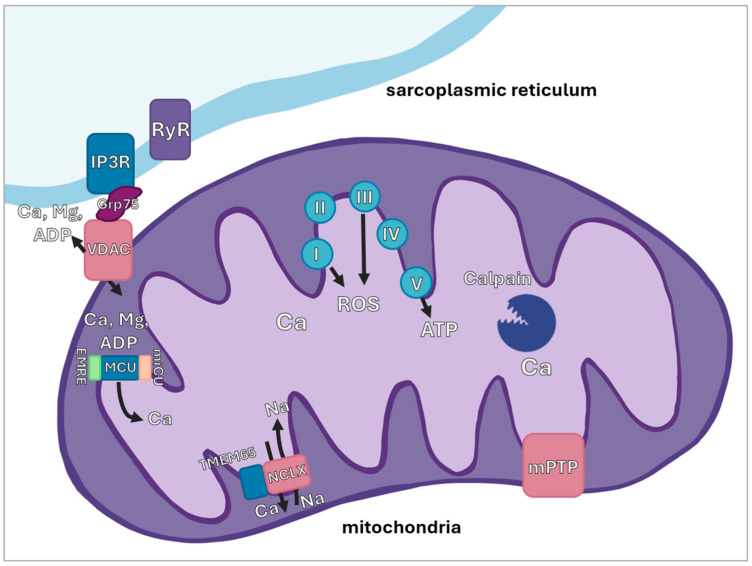
Mitochondrial calcium transport in skeletal muscle. Mitochondria are tethered near the sarcoplasmic reticulum (SR) creating SR-mitochondria microdomains. Calcium ions exit the SR through the ryanodine receptor (RyR) channels or inositol trisphosphate (IP3) receptors and elevate calcium levels in microdomains. Calcium ions pass through the voltage-dependent anion channels (VDAC) in the outer membrane, which allow ions to move freely into the intermembrane space. Entry of calcium into the mitochondrial matrix is primarily mediated by the mitochondrial calcium uniporter (MCU) complex. Chronic or excessive calcium entry (e.g., during muscle unloading) triggers activation of calpains (calcium-dependent proteases) and high ROS levels. Mitochondrial calcium efflux is carried out primarily via the Na^+^/Ca^2+^/Li^+^ exchanger (NCLX) and the mitochondrial permeability transition pore (mPTP).

As previously discussed, intramitochondrial calcium levels play a pivotal role in regulating mitochondrial activity and ensuring the adequate supply of ATP during muscle contraction. Consequently, calcium concentration within the mitochondrial matrix is strictly regulated. According to various reports, mitochondrial calcium levels in resting fast-twitch muscle reach approximately 0.16–0.2 µM; however, our literature search failed to identify data regarding resting mitochondrial matrix calcium in slow-twitch muscles. Given that the ratio of free to bound calcium within mitochondria is reportedly 1:100, the total mitochondrial calcium content may reach tens of millimoles [106,107]. Furthermore, intramitochondrial calcium levels depend not so much on the total concentration of free cytosolic calcium as on the concentration of microdomain calcium in the proximity of sarcoplasmic reticulum (SR)-mitochondria contact sites. Alterations in microdomain calcium concentration, for instance, due to calcium leak from the SR through the ryanodine receptor (RyR) channels or activation of SR inositol trisphosphate (IP3) receptors, can significantly elevate intramitochondrial calcium levels with little to no effect on the overall cytosolic calcium concentration [107]. The IP3 receptors on the SR interact with 75 kDa glucose regulated protein (GRP75), which, in turn, is coupled with the VDAC channel of the outer mitochondrial membrane (see Figure 1 for details).

VDAC is a channel located on the outer mitochondrial membrane; in skeletal muscle, three isoforms are expressed (VDAC1, VDAC2, and VDAC3), which facilitate the passage of a wide range of low-molecular-weight substances, including divalent cations and ADP. VDAC is concentrated on the mitochondrial membrane near contact sites with the SR, in direct proximity to IP3 receptors and ryanodine receptors. This localization enables the transport of microdomain calcium into the mitochondrial intermembrane space [108]. VDAC activity is regulated, in part, by its binding with the chaperone-like protein GRP75 [109] and tubulin. It has been demonstrated that both tubulin polymerization activators and blockers influence the transport of ADP and calcium in mitochondria in the extensor digitorum longus muscle [110].

The inner mitochondrial membrane houses the MCU protein complex, with a molecular weight of approximately 480 kDa, which transports calcium into the mitochondrial matrix. This complex consists of pore-forming subunits (MCU and MCUb) and the essential MCU regulator (EMRE), which interact with regulatory subunits (MICU1, MICU2, and MICU3) [111,112]. The MCU complex facilitates the translocation of calcium cations into the mitochondrial matrix by utilizing the potential difference across the inner membrane, which typically ranges from −160 to −180 mV [113].

The MCU complex interacts with the regulator MICU1, which has two homologous proteins, MICU2 and MICU3. In the presence of MICU1, MICU2 increases the calcium concentration threshold required for MCU-mediated transport, thereby reducing calcium influx into the mitochondrial matrix [114]. Conversely, MICU3 activates MCU complex channels, and the MICU1–MICU3 heterodimer lowers the threshold of MCU sensitivity to calcium [26].

Mechanisms for mitochondrial calcium efflux include the Na^+^/Ca^2+^/Li^+^ exchanger (NCLX), the mitochondrial permeability transition pore (mPTP), and potentially the H^+^/Ca^2+^ exchanger [115,116]. NCLX is expressed in skeletal muscle to a significantly higher degree than in cardiac tissue, which may account for more rapid mitochondrial calcium efflux in skeletal muscle compared to the heart [116]. Recently, the inner mitochondrial membrane protein TMEM65 (transmembrane protein 65) was shown to be essential for NCLX function; however, the regulation of NCLX remains largely uncharacterized [117]. The key regulators of mitochondrial calcium fluxes are shown in Figure 1.

The mPTP complex appears to form a non-specific pore across both the outer and inner mitochondrial membranes, capable of passing molecules up to 1.5 kDa in size [118]. The molecular composition of the pore remains elusive; current hypotheses suggest it may involve the adenine nucleotide translocator (ANT), VDAC [118,119], or even the ATP synthase dimer [120]. Activation of the mPTP can occur in response to calcium overload, ROS excess, or a decline in the proton potential, leading to mitochondrial degradation and, frequently, apoptosis [118]. The primary mechanisms and proteins involved in mitochondrial calcium transport in skeletal muscle are illustrated in Figure 1.

Mitochondrial calcium accumulation in the rat soleus muscle during hindlimb unloading was first reported in 1998 [121]. Accumulation of intramitochondrial calcium, alongside increased mitochondrial ROS generation, has been observed following 24 h of muscle immobilization [83]. In our laboratory, intramitochondrial calcium accumulation was detected after 3 and 7 days of hindlimb unloading in the rat soleus muscle [122,123]. Furthermore, atomic spectroscopy analysis of the mitochondrial fraction revealed a significant increase in calcium content after 15 and 30 days of rat hindlimb unloading [121].

The mechanisms underlying mitochondrial calcium accumulation appear to be linked to the regulation of myoplasmic calcium under conditions of mechanical muscle unloading. An accumulation of calcium within the myoplasm of the mouse soleus is observed as early as the second day of unloading [124] and remains elevated at least until 14 days of rat hind limb unloading [125]. In the human soleus muscle, increased levels of phospho-Ca^2+^/calmodulin-dependent protein kinase II (p-CaMKII), a marker of myoplasmic calcium accumulation, have been reported following 6 and 21 days of dry immersion [48,49]. Our laboratory has demonstrated that preventing myoplasmic calcium accumulation using an L-type calcium channel blocker restored intramitochondrial calcium to control levels [122]. These findings suggest that the elevation of myoplasmic calcium during unloading contributes significantly to mitochondrial calcium accumulation.

Furthermore, a recent review has shown that mitochondria in skeletal muscle may serve as a calcium buffer, regulating myoplasmic calcium levels within certain physiological limits [126]. Consequently, disruptions in calcium-dependent signaling in skeletal muscle may affect mitochondria before an accumulation of calcium can be detected [126]. Based on experimental data, it is hypothesized that the role of mitochondria as a calcium buffer is prominent primarily in slow-twitch muscle fibers, which are most susceptible to the effects of mechanical unloading [126].

**Figure 2:**
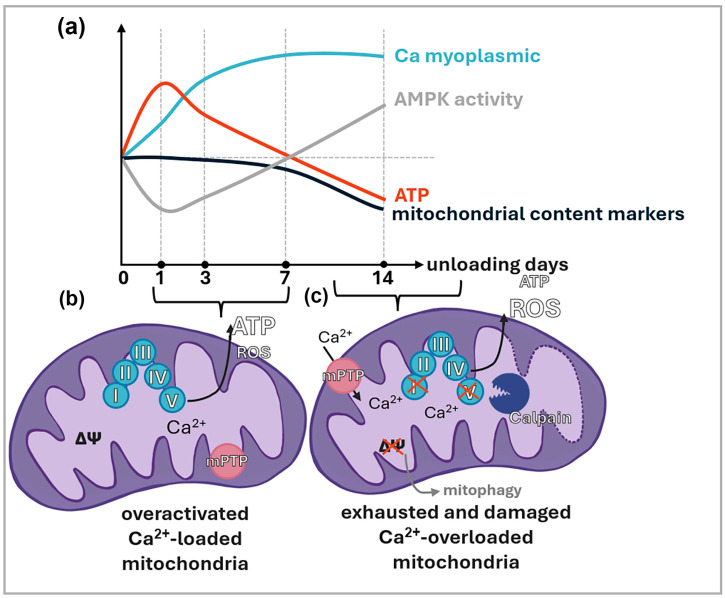
A hypothesized two-phase model of mitochondrial dysfunction in the slow-type muscle during mechanical unloading: (**a**) Time-course changes in mitochondrial activity regulators in the slow-type soleus muscle; (**b**) First stage: mitochondria are loaded by calcium and produce ATP and ROS; (**c**) Second stage: mitochondria experience severe calcium overload, which triggers the activation of intramitochondrial calpains. This leads to the degradation of electron transport chain (ETC) proteins, structural damage to the cristae, and a significant disruption of ATP production.

Pharmacological inhibition of mitochondrial ROS, as well as the pharmacological stabilization of calstabin (which prevents calcium leak through ryanodine receptors), resulted in a reduction in mitochondrial calcium content during 7-day rat hindlimb unloading [43,123]. It appears that under conditions of mechanical unloading, ROS are capable of inducing intramitochondrial calcium accumulation, a process similarly driven by ryanodine receptor dysfunction. The dynamics of calcium and ROS content during postural muscle unloading is graphically shown in Figure 2a. The articles on which Figure 2 is based on are represented in Table 1.

Notably, the prevention of intramitochondrial calcium accumulation was accompanied by a reduction in oxygen consumption by ETC enzymes [123]. Given the previously mentioned non-linear dynamics of ATP content in skeletal muscle during mechanical unloading (characterized by ATP accumulation above control levels on days 1–3), it is plausible that intramitochondrial calcium accumulation contributes to both ATP and ROS accretion during the early stages of unloading via the activation of ETC enzymes. This is indirectly supported by the fact that pharmacological reduction in myoplasmic calcium (which is closely linked to intramitochondrial calcium levels) on day 3 prevents ATP accumulation, inhibits ROS growth, and prevents AMPK inactivation [105,122]. Furthermore, the administration of metformin, an ETC complex I inhibitor, during 3 and 7 days of hindlimb unloading also promotes a reduction in ATP levels and activates AMPK in rat soleus muscle [127,128]. Thus, it is highly probable that calcium-dependent activation of ETC enzymes during the initial stages of mechanical unloading leads to an ATP surplus, which in turn triggers AMPK inactivation and downregulates AMPK-dependent genes, specifically those regulating mitochondrial biogenesis. However, from day 7 of mechanical unloading onward, there is a decline in ATP content and subsequent AMPK activation, occurring alongside elevated intramitochondrial calcium and reduced mitochondrial density. Despite AMPK’s role as a primary activator of mitochondrial biogenesis, its activation during the later stages of unloading fails to prevent the decline in mitochondrial density. The dynamics of ATP accumulation and AMPK activity during unloading are illustrated in Figure 2a. The studies upon which this figure is based are summarized in Table 1.

Unfortunately, the factors driving the changes in ATP content in the soleus muscle from day 7 of mechanical unloading onward remain unknown. Nevertheless, it is established that the reduction in mitochondrial DNA content, a key marker of mitochondrial density, becomes evident specifically starting from day 7 of the unloading. It appears that the alterations in the mRNA expression of mitochondrial biogenesis regulators require this duration to manifest at the protein level and shift the balance between biogenesis and mitophagy sufficiently to impact mitochondrial density. Presumably, from this point forward, the calcium-mediated activation of ETC enzymes can no longer compensate for the overall decline in the quantity of these enzymes. Furthermore, a reduction in mitochondrial density, coupled with an undiminished calcium flux into the mitochondria, likely leads to a further surge in intramitochondrial calcium concentration. Such a prolonged elevation of intramitochondrial calcium in muscle tissue may trigger the activation of intramitochondrial calpains. Specifically, the activation of mitochondrial calpains in Duchenne muscular dystrophy has been shown to induce the proteolysis of respiratory chain complex I and ATP synthase, as well as trigger the opening of the mPTP and the activation of mitophagy [129,130]. These events, in turn, lead to a reduction in ATP synthesis and mitochondrial proton potential. Notably, while AMPK serves as a regulator of mitochondrial biogenesis, it also functions as an activator of mitophagy. Specifically, mitochondrial-associated AMPK in skeletal muscle can initiate mitophagy in response to a localized decline in proton potential [131,132]. Thus, it is probable that after 7–14 days of mechanical unloading in rats (and potentially after 21 days of unloading in humans), the activation of mitochondrial calpains results in diminished mitochondrial proton potential. This, in turn, triggers AMPK activity and subsequent mitophagy, ultimately leading to impaired mitochondrial ATP production.

A hypothesized two-phase model of mitochondrial dysfunction in the slow-type postural muscle during mechanical unloading is presented in Figure 2b,c. According to this hypothesis, muscle unloading occurs in two stages. In the initial stage, mitochondria are activated by calcium and produce excessive ATP in resting muscle, although they may already be sufficiently compromised to become dysfunctional during activity. In the second stage, mitochondria become overloaded with calcium and damaged by calpains, leading to a failure in ATP production even under resting conditions.

Another potential explanation for the observed non-linear ATP dynamics may relate to changes in ATP consumption within the unloaded soleus. During unloading, the electromyographic activity of the soleus muscle shifts non-linearly: while it drops nearly to zero on the first day, it gradually recovers by day 14, eventually exceeding control levels [133]. It is still unclear whether this activity results in increased ATP consumption by muscle fibers. Pharmacological inhibition of this spontaneous activity paradoxically resulted in downregulated ATP content [134]. An additional mechanism potentially underlying the initial ATP accumulation is the myosin transition from the contraction-ready disordered-relaxed (DRX) state to the super-relaxed (SRX) state. This transition could significantly reduce ATP consumption in the resting muscle [135]. However, the impact of this transition on ATP levels in unloading muscle remains to be elucidated.

Another mechanism contributing to altered ATP consumption during muscle unloading involves Na,K-ATPase activity [136]. Since this enzyme is inhibited in the soleus as early as 6–12 h after the onset of hindlimb suspension [104], the initial accumulation of ATP may be driven by reduced ATP consumption by Na,K-ATPase. One more enzyme that consumes ATP in resting muscle is SERCA [137]. Several authors suggest that it is also inactivated at the early stage of muscle unloading; if so, it could also contribute to ATP accumulation in unloaded muscle [105,138].

**Table 1:** Comparative literature data on ATP/AMPK, ROS, calcium and mitochondrial content markers in soleus muscle under dry immersion (DI) in humans and hindlimb suspension (HS) in rodents.

Unloading Model and Time Point	AMPK/ATP	Calcium Markers	ROS	Mitochondrial Content Markers
1 day or less, rodent HS			[82]	[50] downregulation/no changes
	[50,93,139,140]	No data	upregulation	
	downregulation			
1 day or less, human DI	No data	No data	No data	No data
3 days, rodent HS	[92] downregulation	[124] upregulation	[60] upregulation	[60] downregulation
3 days, human DI	[100]	No data	No data	[56] downregulation/no changes
	downregulation			
7 days, rodent HS	[94] no changes	[124] upregulation	[60] upregulation	[141] downregulation
7 days, human DI	[48] no changes	[48] upregulation	No data	[48] downregulation
14 days or more, rodent HS	[94,95,96,97,98,99,101,102] upregulation/contradictory	[124,142] upregulation	[60] upregulation	[59,60] downregulation
14 days or more, human DI	[49] upregulation	[49] upregulation	No data	[49] downregulation

## 4. Mitochondria-Related Mechanisms Implicated in the Loss of Muscle Mass and Function Under Unloading Conditions

### 4.1. Mitochondria and the Regulation of Muscle Mass

**Figure 3:**
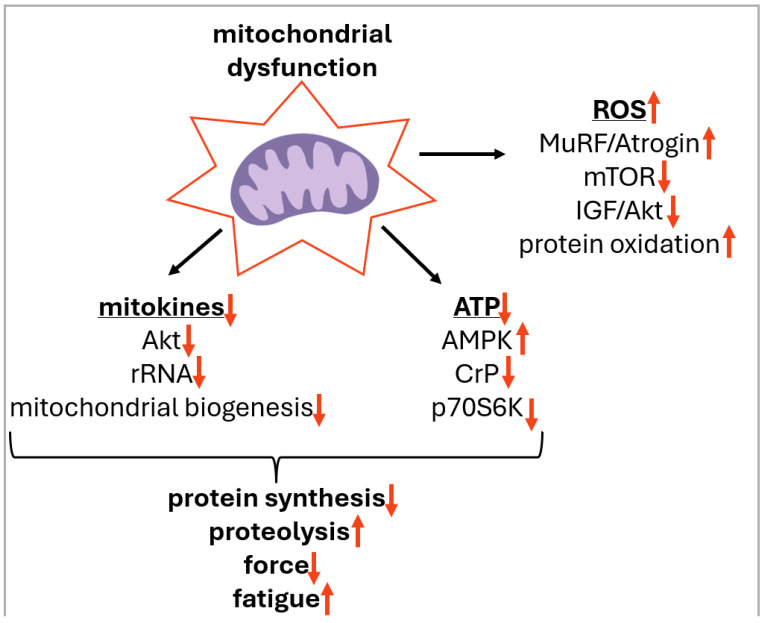
Mitochondrial mechanisms driving postural muscle atrophy and dysfunction under conditions of mechanical unloading.

Mitochondrial dysfunction can contribute to unloading-induced skeletal muscle atrophy by at least four mechanisms: (1) increased mitochondrial ROS production; (2) release of proapoptotic factors from mitochondria, (3) mitochondrial damage or excessive fission leading to reduced ATP production, (4) decreased production of mitokines (see Figure 3 for the details).

Although mitochondrial ROS act as physiological signaling molecules in muscle homeostasis, excessive ROS impairs the pathways that maintain muscle mass. According to the findings of multiple studies, mitochondria have been identified as a primary source of ROS during periods of prolonged disuse [143,144,145]. In line with this, treatment with mitochondria-targeted antioxidants prevents inactivity-induced oxidative stress in skeletal muscle fibers [143,144,145], supporting the view that mitochondria are a dominant ROS source during extended inactivity. On the other hand, it has been demonstrated that the deactivation of mitochondrial ROS does not prevent atrophy associated with rodent hindlimb unloading [146]. Furthermore, in some studies, the prevention of atrophy was not accompanied by the mitigation of ROS accumulation during unloading [123]. In a recent review, it has been suggested that mitochondrial ROS during muscle disuse could trigger protective mechanisms, and disuse-induced mitochondrial remodeling might represent a functional adaptation to recalibrate ATP supply in response to reduced skeletal muscle energy demand [147]. These findings cast doubt on the pivotal role of mitochondrial ROS as a primary regulator of muscle atrophy. Nevertheless, it is certain that mitochondrial ROS make a specific contribution to the progression of atrophic processes.

Chronically elevated mitochondrial ROS may promote muscle wasting by inhibiting protein synthesis and accelerating proteolysis. Oxidative stress, defined as an imbalance between ROS and antioxidants, activates all major proteolytic systems, including calpains, the ubiquitin–proteasome system (UPS) (including ubiquitin ligases muscle RING-finger protein-1 (MuRF1) and Atrogin-1) via activation of p38 MAPK [148], autophagy, and caspase-3. Oxidative stress has been demonstrated to enhance proteolysis through three independent mechanisms. First, oxidative stress often raises the level of free cytosolic Ca^2+^, and elevated cytosolic Ca^2+^ activates calpains and caspase-3 [149,150]. Second, redox disturbances activate transcriptional regulators that upregulate proteolysis-related genes (atrogenes) [151]. Third, oxidative modification of muscle proteins increases their susceptibility to degradation by calpains, caspase-3, and the UPS [152,153]. In addition to accelerating proteolysis, oxidative stress depresses protein synthesis by reducing phosphorylation of translation initiation factor 4E-binding protein 1 (4E-BP1) and impairing mechanistic target of rapamycin (mTOR) complex assembly, thereby blocking translation initiation [154,155].

Beyond increased ROS, damaged mitochondria release mitochondria-specific signaling molecules that activate atrophy pathways. The release of apoptosis-inducing factor (AIF) and cytochrome c into the cytosol activates caspase-3, promoting actin–myosin breakdown and triggering myonuclear apoptosis, which reduces the transcriptional capacity of myofibers [30]. AIF and cytochrome c can be released through mitochondrial outer membrane permeabilization or by opening of the mitochondrial permeability transition pore (mPTP). The mPTP opening is largely regulated by matrix Ca^2+^ and is induced by Ca^2+^ overload [84].

Dysfunctional mitochondria may also contribute to disuse-induced muscle atrophy by impairing oxidative phosphorylation, which reduces ATP production and raises the cellular AMP/ATP ratio. Increased AMP activates AMPK, which can initiate atrophic signaling in part by activating the transcription factor FoxO3 [156,157]. FoxO3 has been shown to promote muscle wasting by upregulating atrogenes that drive proteolysis through both the UPS and autophagy [158,159]. Furthermore, AMPK has been shown to negatively regulate the key anabolic mTOR/70 kDa ribosomal protein S6 kinase (p70S6K)-dependent signaling pathway and protein synthesis in skeletal muscles [160,161]. Importantly, AMPK activation during unloading is context-dependent and can vary according to the stage of unloading (early vs. late), muscle fiber type (slow-twitch vs. fast-twitch), and the experimental model used (hindlimb unloading vs. mechanical ventilation).

In the early 2000s, biologically active peptides released by mitochondria during intense muscular activity were identified and termed mitokines or mitochondria-derived peptides (MDPs); more recent literature also refers to them as mitochondria-derived microproteins [162]. Notably, the term ‘mitokine’ encompasses both MDPs and nuclear-encoded peptides, such as fibroblast growth factor 21 (FGF21) and growth differentiation factor 15 (GDF15), which are expressed in response to mitochondrial protein unfolding. FGF21 has been shown to induce fasting-mediated atrophy and neuromuscular junction disruption under muscle denervation [163,164], while GDF15 contributes to atrophy progression under cancer cachexia and mechanical ventilation [165].

The gene for the most prominent mitokine, mitochondrial open reading frame of the 12S rRNA type-c (MOTS-c), is encoded within mitochondrial ribosomal RNA. Its expression occurs from the same gene as mitochondrial 12S rRNA, but via an alternative reading frame shift [166]. The genes for mitochondrial-derived peptides—humanin and humanin-like peptides in humans, as well as rattin in rodents—are encoded within the mitochondrial 16S rRNA sequence [162].

These peptides function by inactivating pro-apoptotic factors and stimulating cellular antioxidant defense mechanisms [167,168,169]. Mitochondrial stress-regulated MOTS-c has been shown to inhibit the folate cycle, thereby causing purine biosynthesis in the muscle, which leads to activation of AMPK and enhances mitochondrial biogenesis [166]. Upon metabolic stress (glucose restriction), MOTS-c has been demonstrated to translocate to the nucleus, where it contributes to the stress response by regulating gene expression, including genes associated with antioxidant defenses [170]. The content of the MOTS-c peptide declines in both skeletal muscle and systemic circulation during aging and following 7 days of rodent hindlimb unloading [54,171]. In humans, MOTS-c pre-mRNA expression in the soleus muscle is downregulated after 6 days of dry immersion [48]. Notably, transcriptomic analysis indicates that the inactivation of signaling pathways associated with the synthesis of mitochondrial rRNAs (which serve as precursors for MOTS-c mRNA) remains suppressed even after 21 days of dry immersion [49].

It has been demonstrated that MOTS-c administration during muscle immobilization triggers protein kinase B (AKT) activation and inhibits proteolytic signaling [172]. Furthermore, MOTS-c treatment during 7-day hindlimb unloading prevents the reduction in the cross-sectional area of slow-twitch soleus muscle fibers, facilitates the preservation of 18S and 28S ribosomal RNA levels, downregulates proteolytic markers, and maintains fatigue resistance in the soleus muscle [62]. Consequently, mitochondrial dysfunction during mechanical unloading may significantly contribute to muscle atrophy via the suppression of mitokine synthesis.

Mitochondria-related events involved in the loss of postural muscle mass and function under unloading conditions are summarized in Figure 3.

### 4.2. Mitochondria and Regulation of Muscle Function

It is evident that dysfunction of mitochondria (the primary energy suppliers for sustained muscle contraction) can contribute to the increased skeletal muscle fatigability observed under mechanical unloading. Indeed, the heightened fatigability associated with nearly all forms of mechanical unloading is accompanied by a decline in mitochondrial biogenesis, as well as a reduction in mitochondrial protein and DNA content. Conversely, pharmacological agents aimed at restoring mitochondrial biogenesis have been shown to prevent an increase in muscle fatigue [173]. Notably, however, agents that have demonstrated significant efficacy in preserving muscle function, such as beta-guanidinopropionic acid (β-GPA) or metformin, act as inhibitors of mitochondrial respiration while simultaneously activating mitochondrial biogenesis [174,175]. Had mitochondrial respiration been the limiting factor, the administration of respiratory chain inhibitors would likely have further impaired muscle performance. This suggests that during active muscle contraction, mitochondrial quantity and, consequently, the expression levels of biogenesis regulators may be more critical for operational efficiency than the potential inhibition of ETC enzymes by metformin or β-GPA. Furthermore, the administration of agents that reduce intramitochondrial calcium levels during mechanical unloading also contributes to the prevention of increased muscle fatigability. This mechanism may be linked to the mitigation of mitochondrial calcium overload, which can cause mitochondrial damage under unloading conditions [122,123]. Mitochondrial calcium overload not only compromises organelle structure but also drives pathological ROS emission. These reactive species decrease the calcium sensitivity of contractile proteins, ultimately diminishing the muscle’s resistance to fatigue [176].

The contribution of mitochondrial functions to maximum muscle strength during mechanical unloading is difficult to quantify, as mitochondrial activity can modulate both muscle fiber size and, through the regulation of proteolysis, the integrity of the muscular cytoskeleton. The oxidation of sarcomeric proteins, driven by elevated ROS, results in the downregulation of muscle force and compromised contractile performance [176]. Furthermore, mitochondria are involved in the calcium-dependent regulation of skeletal muscle. Given that all these factors are critical for maximum strength generation, their complex interplay complicates an isolated assessment of the mitochondrial role.

## 5. Promising Therapeutic Strategies to Reverse Disuse-Induced Mitochondrial Impairment in Skeletal Muscles

Exercise has been identified as a highly effective therapeutic approach for enhancing mitochondrial content, oxidative phosphorylation quality, and respiratory capacity per mitochondrion [177,178,179]. Exercise preconditioning (an exercise training performed before the onset of disuse) has emerged as a promising non-pharmacological strategy for preventing unloading-induced loss in skeletal muscle mass and function. Fujino et al. (2009) demonstrated that a single bout of 25 min on the treadmill, administered immediately prior to two weeks of hindlimb unloading, significantly mitigated the unloading-induced decline in fatigue resistance and myofibrillar protein content in the soleus muscle of rats [180]. However, the study did not assess markers of mitochondrial or oxidative stress. Subsequently, Theilen et al. (2018) revealed that in the soleus muscle of mice subjected to a concurrent treadmill exercise program (endurance-style exercise plus higher-intensity interval sprint style of exercise) for two weeks prior to seven-day hindlimb unloading, there was lower oxidative stress, as measured by dihydroethidium staining, along with a concomitant increase in antioxidant levels of mitochondrial superoxide dismutase (SOD)-1 and SOD-2 gene expression, as compared to unloading alone [141]. The enhanced antioxidant capacity and the diminished levels of free radicals appear to offer protection to mtDNA, consequently resulting in a reduced incidence of mtDNA mutations. This phenomenon coincided with the maintenance of the expression of markers associated with mitochondrial biogenesis and function [141]. Furthermore, a two-week exercise training period preceding a seven-day period of unloading resulted in the mitigation of soleus muscle weight and fiber cross-sectional area loss [141]. In a separate study, the administration of moderate treadmill exercise on a daily basis for a period of seven days prior to hindlimb unloading was found to offer a protective effect against unloading-induced atrophy in murine gastrocnemius muscle, extending up to seven days of hindlimb unloading. This exercise-induced muscle protection was accompanied by the normalization of the key markers of mitochondrial dynamics (i.e., the balance between mitochondrial fusion and fission) [42]. Nonetheless, the protective effect of seven-day treadmill exercise was observed to dissipate by the 14th day of hindlimb unloading, indicating that a more protracted or vigorous exercise training is requisite to safeguarding skeletal muscles during later stages of unloading [42].

In addition to physical exercise, the administration of mitochondria-targeted nutrients and antioxidants that possess the capacity to neutralize excessive ROS production may serve to reverse mitochondrial dysfunction-induced skeletal muscle impairment under unloading conditions. Liu et al. (2014) have demonstrated that the administration of a combination of mitochondrial nutrients (α-lipoic acid, acetyl-L-carnitine, hydroxytyrosol, and CoQ10) during 3-week hindlimb unloading promoted mitochondrial biogenesis, improved mitochondrial function, and ameliorated the oxidative stress and apoptosis in rat skeletal muscle [181]. A mitochondria-targeted peptide antioxidant, SS-31 (also known as Elamipretide), has demonstrated encouraging outcomes in the mitigation of mitochondrial dysfunction in skeletal muscles under conditions of disuse. A daily subcutaneous injection of SS-31 to rodents during 7- or 14-day hindlimb cast immobilization attenuated disuse-induced increases in mitochondrial ROS emission, as well as prevented oxidative stress, protease activation, and myofiber atrophy in soleus muscle [145]. In addition, a study on human subjects revealed promising outcomes with the administration of SS-31. A 5-day treatment of patients with primary mitochondrial myopathy with SS-31 resulted in increased exercise performance, as indicated by an increase in the distance walked in the 6 min walk test [182]. Furthermore, SS-31 has recently received approval for medical use in the USA as the first treatment for Barth syndrome (a rare X-linked mitochondrial disease) (https://www.fda.gov/news-events/press-announcements/fda-grants-accelerated-approval-first-treatment-barth-syndrome, accessed on 21 January 2026). The results from human studies indicate that SS-31 may be a promising pharmaceutical agent for the treatment of disuse-induced muscle atrophy associated with mitochondrial dysfunction. As demonstrated in the study by Sun et al. (2021), the administration of astaxanthin, a potent antioxidant, during hindlimb unloading led to the prevention of a decline in muscle mass and cross-sectional area of type I and type IIa fibers in the murine soleus muscle [183]. This intervention successfully mitigated the occurrence of mitochondrial dysfunction, which is often induced by oxidative stress. In particular, astaxanthin exhibited a marked inhibitory effect on the reduction in mitochondrial complexes I and III protein content, while concomitantly enhancing mitochondrial oxidative phosphorylation and biogenesis in the soleus muscle of unloaded mice [183]. Furthermore, astaxanthin treatment has been shown to mitigate the generation of mitochondrial ROS, cytochrome c release into the cytosol, and caspase-3 activation in muscle Sol8 myotubes (cells extracted from the soleus muscle) [183].

A separate study found that treatment of rats with the mitochondrial ROS scavenger mito-TEMPO during one-week hindlimb unloading partly prevented rat soleus muscle atrophy, attenuated increased intramitochondrial calcium and reduced the content of oxidized tropomyosin (a marker of ROS levels in muscle tissue). In addition, mito-TEMPO treatment prevented an unloading-induce decrease in the expression of the key markers of mitochondrial biogenesis [43]. The daily administration of mitokine MOTS-c (a short peptide of mitochondrial origin) during 7-day hindlimb unloading prevented slow-to-fast fiber type transformation in rat soleus, improved fatigue resistance, and attenuated fiber atrophy in slow-type fibers [62]. Natural polyphenols, such as resveratrol and curcumin, and the anti-diabetic drug metformin (an AMPK activator), have also demonstrated efficacy in protecting slow-type rat soleus muscle from deleterious functional, metabolic, and morphological changes associated with mechanical unloading, partly through activating antioxidant defense systems [127,184,185,186].

Another promising strategy to reverse mitochondrial impairment in skeletal muscle is mitochondrial transplantation therapy (MTT). This strategy involves delivering functional mitochondria to dysfunctional muscles to restore energy production and improve muscle function. Two primary mitochondrial transplantation strategies are currently under investigation: (1) direct administration of isolated mitochondria; (2) the use of extracellular vesicles (EV). EV are lipid-bound vesicles released by various cell types and play a crucial role in tissue crosstalk by transporting and delivering bioactive molecules and mitochondria [187]. Furthermore, mitochondrial transplantation from stem cells shows promise as a treatment for skeletal muscle atrophy, with mesenchymal stem cells (MSCs) being the most promising candidates for this approach [188]. Stem cell-based mitochondrial transplantation can be performed in two ways: either by directly transplanting stem cells into patients or by extracting mitochondria-rich EV from stem cells and implanting them into patients [188]. Preclinical studies and clinical trials have demonstrated the efficacy of stem cell therapy in reducing muscular atrophy and enhancing physical performance and motor function (reviewed in [188]). Mechanistically, the effects of transplanted mitochondria and EV are achieved through the following mechanisms: (1) an enhancement of bioenergetics and redox processes, manifested as an increase in oxidative phosphorylation and ATP production; (2) the maintenance of redox homeostasis via antioxidant pathways involving nuclear factor erythroid 2-related factor 2 (Nrf2), SOD, and forkhead box protein O (FOXO) signaling; (3) the modulation of mitochondrial dynamics via the regulation of fission and fusion; (4) the preservation of mitochondrial homeostasis through the enhancement of mitophagy (increased LC3-II/LC3-I ratio) and mitochondrial biogenesis (PGC-1α and TFAM upregulation) [187]. These mechanisms underpin the therapeutic potential of mitochondrial and EV-based interventions across a range of diseases [187].

Alway and colleagues have recently investigated the effects of MTT on the restoration of skeletal muscle mass and function following BaCl_2_-induced muscle injury [189]. Donor mitochondria were suspended in phosphate-buffered saline and injected into the tail vein of mice that had muscle injury. MTT has been demonstrated to expedite the restoration of neuromuscular force and muscle fiber size, particularly during 7 to 14 days following muscle injury, when compared to control muscles [189]. In addition, Dubinin and colleagues (2024) have investigated the effect of MTT on the development of muscle pathology in dystrophin-deficient *mdx* mice [190]. The systemic intramuscular injections of allogeneic mitochondria result in a decrease in the intensity of oxidative stress in the skeletal muscle mitochondria of *mdx* mice and a reduction in calcium overloading [190]. These effects were accompanied by the normalization of the organelle ultrastructure and SR/mitochondria contact interactions. MTT has been demonstrated to result in enhanced muscle strength, specifically in the grip strength of the hindlimbs, and increased endurance, as evidenced by the running-wheel activity test [190].

Despite the promising nature of the discussed interventions, several limitations must be acknowledged when translating these findings into clinical practice. First, there is a significant gap in data regarding optimal dosage and long-term safety profiles, particularly for pharmacological agents targeting AMPK or ROS. Second, the duration of most studies remains relatively short, leaving the long-term efficacy of these treatments during extended unloading periods uncertain. In addition, the relevance of these interventions varies across different models; for example, the systemic physiological shifts in spaceflight (e.g., fluid shift and radiation) may change drug pharmacokinetics compared to ground-based analogs. Thus, future studies should clarify these issues.

## 6. Conclusions and Future Directions

Mechanical unloading of skeletal muscle induces complex alterations in mitochondrial function that extend beyond simple inactivation. These changes involve biphasic dynamics in ATP content and mitochondrial oxygen consumption, suggesting a two-stage process of mitochondrial dysfunction. Alterations in ATP content, increased ROS production, and downregulation of mitokine expression disrupt muscle proteostasis, calcium signaling, cytoskeletal integrity, muscle force, and fatigue resistance. Mitochondrial calcium accumulation appears to be the central mediator of this dysfunction. It is implicated in the first stage (characterized by increased ATP and ROS along with reduced biogenesis) and the second stage (marked by a decline in ATP synthesis).

However, the specific role of calcium overload during long-term muscle unloading warrants further investigation. Future studies should analyze the roles intramitochondrial calpains play in regulating mitochondrial function during muscle disuse, as well as directly assess the level of mitophagy in different muscles and at various time points of disuse. Crucially, key parameters of mitochondrial function, including mitochondrial network integrity, mitochondrial membrane potential (ΔΨ), and the formation of supercomplexes, have yet to be described under conditions of postural muscle mechanical unloading.

Several therapeutic strategies are currently being explored to counteract unloading-induced mitochondrial dysfunction. These include endurance exercise (especially preconditioning prior to unloading), various antioxidants, mitokine injections, substances targeting mitochondrial calcium overload, and novel approaches like mitochondrial transplantation.

An intriguing direction for future studies would be to investigate the interplay between mechanical unloading and underlying genetic factors. Specifically, understanding how genomic instability, such as transposable element activation [191], or how specific mutations in genes like WFS1 [192,193] influence the rate of muscle atrophy could provide a more comprehensive picture of individual susceptibility to disuse-induced wasting. Furthermore, exploring the overlap between mitochondrial gene variants associated with neurodegeneration [194] and those affecting postural muscle integrity may help refine the boundaries between primary muscular dystrophy and secondary atrophy.

## Data Availability

No new data were created.

## Abbreviations

The following abbreviations are used in this manuscript:

4E-BP1	eukaryotic initiation factor 4E binding protein
p70S6K	70 kDa ribosomal protein S6 kinase
AMPK	AMP-activated protein kinase
ACC	acetyl-CoA carboxylase
IGF	Insulin-like growth factor
AKT	protein kinase B
ATP	adenosine triphosphate
ADP	adenosine diphosphate
AMP	adenosine monophosphate
CrP	Creatine phosphate
HS	hindlimb suspension
HU	hindlimb unloading
MuRF	muscle RING-finger protein
MCU	mitochondrial calcium uniporter
MyHC	Myosin heavy chain
mTOR	mammalian/mechanistic target of rapamycin
ROS	reactive oxygen species
ETC	electron transport chain
VDAC	voltage-dependent anion channel
COX7RP	cytochrome c oxidase assembly factor
OPA1	optic atrophy 1/mitochondrial dynamin-like 120 kDa protein
TOMM20	translocase of the outer mitochondrial membrane 20
LAMP1	lysosomal-associated membrane protein 1
PGC-1α	peroxisome proliferator-activated receptor gamma coactivator 1-alpha
TFAM	mitochondrial transcription factor A
Fis1	mitochondrial fission 1 protein
Drp1	dynamin-related protein 1
COX-1	cytochrome c oxidase 1
SERCA	Sarcoplasmic/Endoplasmic Reticulum Ca^2+^ ATPase
NADPH	nicotinamide adenine dinucleotide phosphate
NOX2	NADPH oxidase 2
SR	sarcoplasmic reticulum
UPS	ubiquitin-proteasome system
JAK	Janus kinase
STAT3	signal transducer and activator of transcription
IP3	inositol trisphosphate
GRP75	75 kDa glucose regulated protein
EMRE	essential MCU regulator
NCLX	Na^+^/Ca^2+^/Li^+^ exchanger
mPTP	mitochondrial permeability transition pore
ANT	adenine nucleotide translocator
TMEM65	transmembrane protein 65
CaMKII	Ca^2+^/calmodulin-dependent protein kinase II
AIF	apoptosis-inducing factor
MOTS-c	mitochondrial open reading frame of the 12S rRNA type-c (mitokine)
β-GPA	beta-guanidinopropionic acid
MTT	mitochondrial transplantation therapy
SOD	superoxide dismutase
EV	extracellular vesicles
MSCs	mesenchymal stem cells
Nrf2	nuclear factor erythroid 2-related factor 2
FOXO	forkhead box protein O
FGF21	fibroblast growth factor 21
GDF15	growth differentiation factor 15
